# The Structure, Classification, Functional Diversity and Regulatory Mechanism of Plant C2H2 Transcription Factors

**DOI:** 10.3390/biology15060471

**Published:** 2026-03-14

**Authors:** Junbai Ma, Xinyi Zhang, Shan Jiang, Shuoyao Fei, Lingyang Kong, Meitong Pan, Wei Ma, Weichao Ren

**Affiliations:** College of Pharmacy, Heilongjiang University of Chinese Medicine, Harbin 150040, China; 15114516116@163.com (J.M.); zhangxinyi121102@163.com (X.Z.);

**Keywords:** C2H2 transcription factor, plant growth and development, biological function, regulatory mechanism

## Abstract

Cys2/His2-type zinc finger transcription factors (C2H2 TFs) constitute one of the largest and most functionally diverse transcription factor families in plants, playing core regulatory roles in multiple aspects of plant growth, development, and stress adaptation. Based on literature data from databases including PubMed (1995–April 2026) and integrated with bioinformatics analyses, this review provides a comprehensive overview of this family. We first summarize the structural characteristics and classification systems of C2H2 TFs, and elucidate their evolutionary dynamics from lower plants to angiosperms. Regarding their impact on plant organ development, beyond key biological processes, this review details the molecular mechanisms of C2H2 TFs in floral organ morphogenesis (e.g., petal, sepal, stamen, and ovule development), pollen fertility maintenance, and flowering time regulation. Concurrently, we systematically analyze their functional pathways in responses to abiotic stresses (drought, high salinity, low temperature, aluminum toxicity, etc.) and biotic stresses (pathogens, pests), clarifying the molecular networks through which they coordinate reactive oxygen species (ROS) homeostasis, stomatal movement, and osmotic regulation by modulating hormone signaling pathways such as ABA, SA, and JA. Furthermore, this review discusses major limitations of current research, including knowledge gaps concerning functional redundancy, pseudogenization phenomena, and cell type-specific regulation. We also provide perspectives on future research directions leveraging cutting-edge technologies such as CRISPR gene editing, single-cell sequencing, and multi-omics integration, as well as their application prospects in crop stress resistance breeding and quality improvement. This review provides ideas for in-depth research on the regulatory network and related functions of C2H2 TFs, and offers reference value for improving plant traits, enhancing plant resistance, and increasing the production of plant secondary metabolites.

## Simple Summary

Cys2/His2-type zinc finger transcription factors (C2H2 TFs) constitute one of the largest transcription factor families in plants and play critical regulatory roles in growth, development, and stress responses. This review systematically summarizes the structural characteristics, classification systems, and evolutionary dynamics of this family, with a focus on their functional mechanisms in flower development, abiotic stress responses (drought, salt, low temperature, aluminum toxicity and so on), and biotic stress responses. We elucidate the molecular networks through which C2H2 TFs integrate hormone signals such as ABA to regulate reactive oxygen species homeostasis and stomatal movement. Through cross-species comparative analysis, we examine the evolutionary differences between woody and herbaceous plants and discuss current research limitations as well as future application prospects based on gene editing and multi-omics technologies. This review provides a theoretical reference for crop stress resistance breeding and quality improvement.

## 1. Introduction

During the growth and development of plants, they are exposed to various biological and non-biological stresses. Therefore, under long-term stress, a finely regulated genomic regulatory network gradually evolves in plants, enabling them to maintain their growth, development, and survival [1]. Transcription factors (TFs), the core molecules regulating gene expression, play an indispensable role in plants’ adaptation to environmental changes [2]. In recent years, with the rapid development of genomic, transcriptomic, metabolomic and proteomic technologies, C2H2 TFs, is one of the widely existing and functionally diverse transcription factor families in plants; their members regulate the transcription of target genes by specifically binding to DNA sequences and participate in plant growth and development, hormone signal transduction, secondary metabolite synthesis, and responses to environmental stress [3].

**Figure 1:**
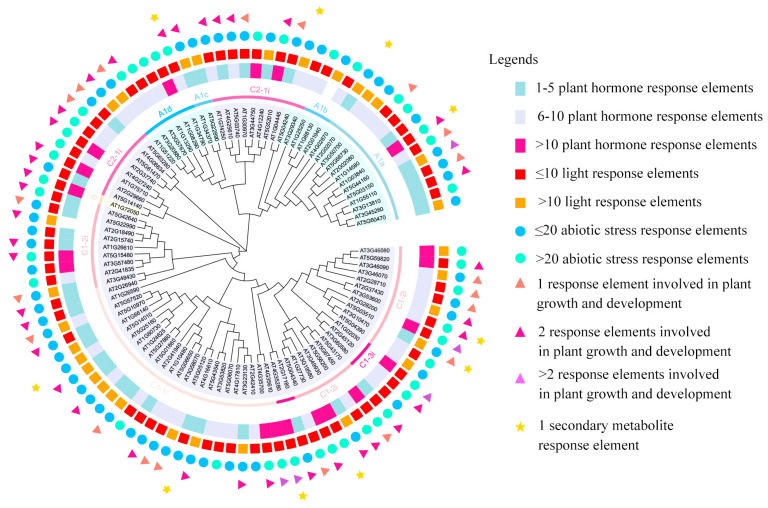
Phylogenetic analysis and promoter cis-element prediction of the *A. thaliana* C2H2 transcription factor family. A neighbor-joining (NJ) phylogenetic tree was constructed based on the protein sequences of all members of the *A. thaliana* C2H2 TFs. Members were classified according to the distribution patterns of their zinc finger domains. Cis-acting elements within the 2000 bp upstream region of the ATG start codon for each transcription factor were predicted. Different colors and shapes represent the types and abundances of these cis-elements, with corresponding annotations provided in the legend on the right.

The core structure of the members of C2H2 TFs comprises highly conserved Cys2/His2 zinc finger motifs. Each zinc finger motif binds to Zn^2+^ through coordination bonds, forming a stable β-β-α three-dimensional conformation, which specifically recognizes and binds to the specific nucleotide sequences in the major groove of the DNA double helix [4]. Typically, the zinc finger domains of C2H2 TFs consist of multiple tandemly repeated zinc finger units. These units’ number, arrangement, sequence, and spatial conformation jointly determine the specificity of their DNA binding patterns and the diversity of target gene transcriptional regulation, endowing members of this family with complex and precise regulatory functions in the gene expression network [5]. Moreover, C2H2 TFs have extensive functions in plants, regulating plant organ growth and development as well as secondary metabolite synthesis. They can also respond diversely to stress responses. Some characteristic TFs and their homologous genes in *Arabidopsis thaliana* (*A. thaliana*) have significant effects on flower development, and we have emphasized this point in the article. Figure 1 presents the grouping of the C2H2 TFs in *A. thaliana* and the combined analysis of cis-acting regulatory elements. We found that C2H2 TFs can be involved in plant growth and development, the synthesis of plant hormones, responses to abiotic stress, and the regulation of secondary metabolite synthesis. Therefore, conducting a comprehensive review of plant C2H2 TFs is necessary, as it can provide a more comprehensive perspective for in-depth molecular biology research.

Cross-species comparative analysis reveals significant differences in the evolutionary trajectories of C2H2 TFs between woody and herbaceous plants. Our findings indicate that woody plants possess a higher proportion of plant-specific Q-type C2H2-ZFPs (containing the QALGGH motif), with poplar exhibiting a proportion of 57%, significantly higher than that in *A. thaliana* (36%) and *Oryza sativa* (*O. sativa*) (34%) [6]. Moreover, woody plants demonstrate more active expansion mechanisms driven by fragment duplication and tandem duplication. In terms of functional differentiation, woody plants have evolved regulatory networks associated with perennial growth habits, including secondary growth and wood formation (e.g., *PagIDD15A* [7]), seasonal dormancy, fruit quality development (e.g., *CitZAT4* [8]), and secondary metabolite biosynthesis (e.g., *AsZFP9* [9]). In contrast, research on herbaceous plants has primarily focused on abiotic stress responses, floral organ development, and pathogen defense. This divergence reflects the evolutionary adaptation of C2H2 TFs to perennial life history strategies.

This article systematically reviews the progress of research on the functions and molecular mechanisms of C2H2 TFs in plants. It was achieved by searching the relevant literature in PubMed (1995–2026, data up to April 2026) with the keywords “C2H2 transcription factors” and “ in plants”, and by using the PlantTFs online tool (http://planttfdb.gao-lab.org/) (accessed on 10 November 2025) to search for C2H2 TFs and study their domains. All C2H2 TFs of *A. thaliana* were downloaded, and their cis-acting response elements were predicted using TBtools II v2.332 (https://github.com/CJ-Chen/TBtools/releases) (accessed on 10 November 2025). Finally, MEME Suite 5.5.9 software (https://meme-suite.org/meme/) (accessed on 10 November 2025) and the biorender online website (https://www.biorender.com/) (accessed on 10 November 2025) were used to draw and beautify the pictures. From multiple perspectives, this article can serve as a practical reference point for understanding the regulatory network and related functions of plant C2H2 TFs, providing valuable insights for applications in resistance breeding and crop improvement.

## 2. The Structure and Classification

C2H2 TFs are widely present in plants and were first discovered in 1985 during biochemical research on the interaction between the transcription factor TFIIIA and 5S RNA in the African clawed frog. Subsequent studies further revealed the three-dimensional structure of zinc fingers and the specific way they interact with DNA. In the 1990s, with the vigorous development of plant genomics, the first C2H2 TF in plants, *ZPT2-1* (*EPF1*), was identified in DNA-binding proteins of petunia. EPF1 can specifically interact with the promoter region of 5-enol-pyruvyl-shikimate-3-phosphate synthase (*EPSPS*). With the continuous evolution of plant species, the C2H2 TFs family has undergone multiple whole-genome duplications, tandem duplications, and functional differentiation events, resulting in a significant increase in its quantity and functional diversity. Although the core zinc finger structure CXCX_3_HX_3_H remains relatively conserved, the non-zinc finger regions (such as transcriptional regulatory domains) exhibit high sequence diversity, thereby driving the functional differentiation of C2H2 TFs [10]. Based on bioinformatics annotation, 17,740 members of C2H2 TFs have been identified from plants. Moreover, C2H2 TFs show significant differences among different species. Among them, *Brassica napus* (*B. napus*), *Glycine max* (*G. max*), and *Gossypium hirsutum* (*G. hirsutum*) are currently the three plants with the most *C2H2* gene family members discovered in plants, with 368, 321, and 318 *C2H2* family members, respectively, being identified. This quantitative advantage may suggest that during the long-term evolutionary process of these species, C2H2 TFs have played more diverse and crucial roles in their complex growth and development regulation, environmental adaptation, and species-specific biological processes. In the model plant *A. thaliana*, only 116 members of the C2H2 TFs were found. This difference reflects that the expansion mechanisms of C2H2 TFs vary among plant species during evolution and environmental adaptation. Through long-term evolutionary selection, the simplicity of the *A. thaliana* genome has retained a relatively small number of core regulatory factors.

From a structural perspective, C2H2 TFs possess a unique β-β-α folding pattern. The Zn^2+^ is coordinated and linked by two cysteine and histidine residues, forming a stable tetrahedral structure. Through interactions with hydrogen donors and acceptors exposed in the central groove, it specifically recognizes and binds to specific nucleotide triplexes [11]. Meanwhile, each zinc finger forms an independent domain, and its stability mainly depends on Zn^2+^ and the hydrophobic core within the structure [10,12]. At the α-helix region, the C2H2 TFs of plants typically possess the conserved structure of QALGGH, which enables them to directly participate in the recognition and binding to the major groove of DNA [13,14]. In addition, C2H2 TFs in plants also contain multiple functional domains. Usually, they have a nuclear localization signal region (NLS), an L-box functional region, and a DLN-box functional region. Studies on genes such as *ZPT2-3*, *STZ*, *CAZFP1*, and *AtZFP11* have shown that the absence of any of these domains can decrease their expression levels or transcriptional activity [15,16]. However, it rarely contains additional multi-polymerization domains or protein interaction domains.

**Figure 2:**
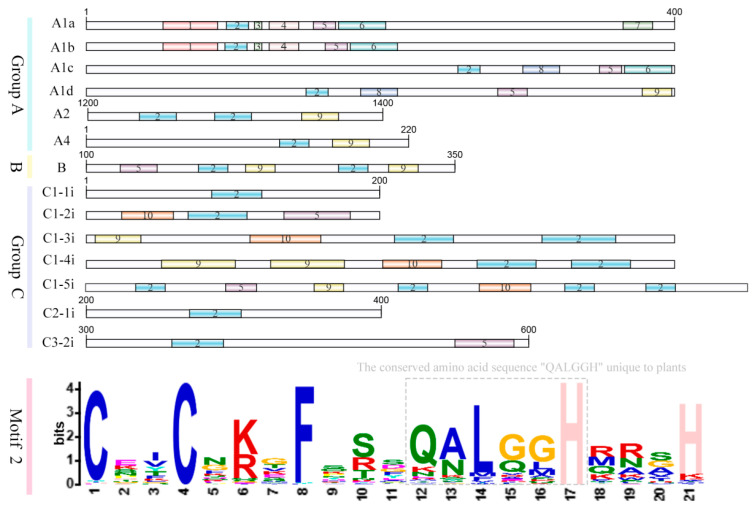
Classification of plant C2H2 TFs based on zinc finger domain distribution patterns. Plant C2H2 TFs are classified into three major groups (A, B, and C) according to the arrangement of their zinc finger domains. Group A features tandemly arrayed zinc fingers with spacers typically fewer than 12 amino acids. Group B exhibits a mixed distribution pattern, while Group C contains dispersed zinc fingers (fewer than five). Group C is further subdivided into C1, C2, and C3 subgroups. All groups harbor the plant-specific QALGGH conserved motif. The classification features of each group and subgroup are indicated by distinct colors and symbols.

In *A. thaliana*, based on the zinc finger distribution pattern, C2H2 TFs are classified into three main groups, including Group A, which contains a tandemly arranged zinc finger array with closely packed zinc fingers and peptide intervals typically less than 12 amino acids. Group B has zinc finger structures distributed in a mixed pattern, and Group C contains dispersed zinc finger structures (fewer than 5). The zinc finger domains usually contain the plant-specific QALGGH conserved motif. According to the difference in the spacing between the two histidine residues in the zinc finger domain, Group C can be further divided into subsets C1, C2, and C3 [17]. The C1 subgroup was further divided into five subgroups based on the number of zinc finger domains [18,19]. The research has found that A1 and C1 belong to a plant-specific family and mainly participate in the transcriptional regulation process. The C2 and C3 subgroups are related to ancient cellular pathways (Figure 2).

Among plant C2H2 TFs, based on the type of zinc finger domain, they are classified into the Q type, which contains the plant-specific conserved amino acid sequence “QALGGH” and the conserved spacer sequence “X2-C-X2-C-X7-QALGGH-X3-H” [20]. The M-type generally contains one or more degraded “QALGGH” motifs, such as ALGGH, LGGH, or variant sequences like AL_GH, and the C-type does not contain any conserved motifs [21].

## 3. The Role in Plant Growth and Development

The formation and development of plant organs is a complex process involving the coordinated action of multiple factors at various levels. As one of the important regulatory factors, C2H2 TFs in plants, jointly with intermediate substances, determine the morphological formation and functional differentiation of organs during multiple stages of plant growth and development, demonstrating functional diversity. The summary of existing studies reveals that C2H2 TFs may precisely control organ growth by establishing a dynamic balance between different hormone pathways, participating in protein modification pathways, and collaborating with other regulatory factors. For instance, in *Gerbera jamesonii*, *GjWIP2* mediates the crossover between Gibberellin (GA), abscisic acid (ABA), and Indole-3-acetic acid (IAA) to inhibit cell proliferation during hybridization, thereby controlling organ growth [22]. In *Zea mays* (*Z. mays*), *ZmDi19-7* physically interacts with the ubiquitin receptor protein ZmDAR1b, and they jointly function in the ubiquitin-proteasome pathway to regulate the cell size in *Z. mays*, thereby influencing the plant height and organ size of *Z. mays* [23].

In this section, we have described that C2H2 TFs play multiple regulatory roles during the growth and development of plants, including influencing the formation of embryos, trichomes, roots, stems, leaves, flowers, seeds, and fruits, the synthesis of secondary cell walls in plants, and the effects on hormones. It is worth noting that many characteristic C2H2 TFs play significant roles in forming and developing plant floral organs through different mechanisms.

### 3.1. The Influence on the Formation of Trichome

C2H2 TFs play a central regulatory role in forming and developing plant trichomes, constituting a complex and orderly genetic regulatory network. They hierarchically regulate trichomes’ initiation, density, elongation, and type differentiation. For instance, in *A. thaliana*, C2H2 TFs such as *GIS*, *GIS2*, *GIS3*, and *ZFP8* jointly participate in the initiation regulation of the trichome structures of floral organs, mainly by upregulating the expression of *GL1*, influencing the timing of trichome formation [24]. Among them, *ZFP8* can regulate the density and length of glandular trichome, while *GIS3* plays a key role in initiating and elongating non-glandular trichome [25]. In *Nicotiana tabacum* (*N. tabacum*), the *GIS* plays a crucial role in the formation of trichomes by integrating the GA metabolic pathway with key transcriptional complexes (such as GL3/EGL3-GL1-TTG1) and cell cycle regulatory factors *SIAMESE* (*SIM*) [26]. Furthermore, *GIS1* and *GIS2* can also function downstream of *SPINDLY* and upstream of *GLABROUS1*, further highlighting their pivotal role in the formation of the trichome structure [27,28]. Functionally, *ZFP5* and *ZFP6* are equivalent to *GIS1/GIS2* and can mediate the coordinated regulation of GA and cytokinin (CTK) on the initiation of the trichome structures through interaction with proteins related to the trichome structures, such as TRY [29,30]. Overexpression can induce ectopic trichome structures, indicating that they are decisive in forming the trichome structure pattern [31,32]. In *Solanum lycopersicum* (*S. lycopersicum*), the *H* and *SPARSE HAIR* (*SH*) gene act as upstream regulatory factors of *SlZFP5*, respectively, regulating the initiation and elongation of type I and type III multicellular trichome structures. Their expression levels directly determine whether the trichome structures can form normally [33].

### 3.2. The Influence on the Development of Roots

By constructing a complex multi-level regulatory network, C2H2 TFs play crucial and diverse regulatory roles in plant root development [34,35]. It participates in the basic process of root morphology formation and can integrate various internal and external signals to achieve precise regulation of root growth. For example, *STOP1* (*AT1G34370.1*) promotes nutrient absorption and regulates the initiation of lateral roots by activating borate channel proteins. At the same time, *WIP1* (*AT1G34790.1*) affects the normal formation of roots by coordinating the direction of cell division during the embryonic stage [36]. *GAZ* plays a dual role in the ABA and GA signaling network, constituting a feedback regulatory loop between these two hormone pathways to coordinately control the timing and extent of middle cortex formation in root tissues [37]. *AtZFP5* can directly activate the transcription of the ethylene signaling core gene *EIN2*, forming a positive feedback loop to regulate trichome initiation and elongation under nutrient stress [38]. On the other hand, *ZAT1* (*AT1G02030.1*), *ZAT4* (*AT2G45120.1*), and *ZAT9* (*AT3G60580.1*) inhibit root growth by relying on conserved EAR motifs [39]. *ZFP1* inhibits root hair development downstream of *GL2* through the GL2/ZFP1/RSL pathway [40]. *ZAT14* regulates the programmed cell death (PCD) of the root cap and promotes PCD of the small column root hairs [41]. This bidirectional regulatory ability enables plants to flexibly adjust their root development strategies in response to environmental changes. C2H2 TFs are also involved in specific developmental processes, such as root nodule formation. For example, in *G. max*, their binding elements are significantly enriched in the root nodule-specific module promoters [42].

### 3.3. The Influence on the Formation of Leaves

During the development of leaves, C2H2 TFs cover multiple aspects, from cell division and polarity establishment to the final shaping of leaf morphology. Among them, the function of the WIP subfamily is particularly prominent, primarily as they precisely regulate the cell expansion and division process of leaves through different modes such as functional redundancy, antagonism, or synergy [17]. For instance, *WIP6* (*AT1G13290.1*) maintains the typical developmental sequence [43]. Moreover, its homologous genes even dominated the development of specialized structures, such as stomata in early land plants like *Marchantia polymorpha* [44]. More importantly, C2H2 TFs are deeply intertwined with the plant hormone signaling network. For example, in *B. napus*, *BnaC06.WIP2* reshapes the distribution of IAA and regulates the levels of ABA and CTK, directly determining and accelerating the formation of lobed leaves [45].

Apart from WIP, C2H2 TFs are also indispensable in establishing the spatial configuration of leaves and the characteristics of the epidermis [46]. In *A. thaliana*, factors such as *JAG* and *OsZFP7* in *O. sativa* coordinate cell division and expansion, jointly maintaining the dorsal–ventral polarity and flattened growth of leaves [47]. While in *Limonium bicolor*, *LbZFP5* can positively promote the normal development of leaf epidermis by inhibiting the expression of key negative regulatory factors (such as *TRY*) [48,49].

### 3.4. The Effects on Buds and Stems

C2H2 TFs also play a crucial role in forming shoot tips and stem morphology. For instance, the expression of *AtZFP1* (*AT1G80730.1*) in *A. thaliana* in meristems, young leaves, and vascular primordia indicates its involvement in the early development process of the shoot tip. It suggests its potential role in top growth and the establishment of leaf vein patterns [50]. C2H2 TFs are also involved in integrating environmental responses and morphological construction, such as *SGR5* (*AT2G01940.1*), which affects the growth direction of inflorescence stems by regulating the gravity perception mechanism [51]. Regarding stem development, factors such as *HVA* (*AT5G27880)* have been proven to regulate the proliferation and differentiation of vascular bundles directly and significantly influence the construction of the vascular system [52]. *RBE* (*AT5G06070*) negatively regulates the formation layer proliferation driven by the TDIF-PXY pathway by directly binding to and inhibiting the *WOX4* promoter, thereby suppressing the secondary growth of the stem [53].

### 3.5. The Influence on the Formation of Flowers

**Figure 3:**
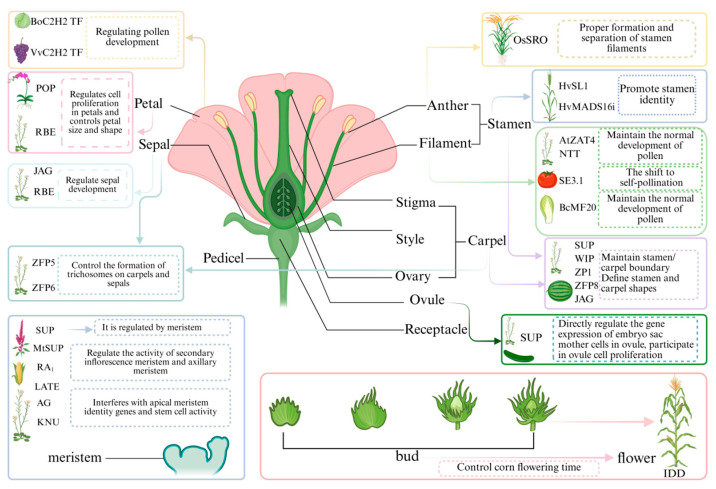
Regulatory roles of C2H2 TFs in flower development.

C2H2 TFs form the core network that regulates plant flower development by regulating downstream target genes and hormone signaling pathways. Their functions span the entire process from flower induction to the maturation of flower organs [54]. In Figure 3, we present the functions of the characteristic C2H2 TFs in all floral organs. Regarding regulating flowering time, C2H2 TFs can determine the optimal flowering time by integrating internal and external signals. For instance, the maize IDD1 can autonomously promote flowering [55]. While FLC in *A. thaliana* acts as a key flowering repressor, its expression is inhibited by the plant-specific C2H2-SET domain protein AtCZS through a non-vernalization pathway, precisely controlling the transition from vegetative growth to reproductive growth [56,57]. *CaZAT5* delays flowering time in *S*. *lycopersicum* and affects pollen viability and anther dehiscence [58].

In forming floral organ morphology, the functions of C2H2 TFs exhibit a high degree of specificity and precision. For instance, *JAG* and *NUBBIN* (*NUB*) work together to shape the specific morphology of stamens and carpels [59]. *SUP* (*AT3G23130.1*) is explicitly expressed in some regions of the floral meristem and is crucial for maintaining the boundary between stamens and carpels, preventing organ fusion. Its function has been observed from *A. thaliana* to *Cucumis sativus* (*C. sativus*), involving the proliferation of boundary cells [60,61]. *RBE* promotes petal growth by relieving the inhibition of miRNA319 on TCP, thereby precisely controlling the size and shape of the petals [62,63,64].

During the process of plant reproduction, pollen can transfer the male gametes into the embryo sac before double fertilization occurs [65,66]. The C2H2 TFs play a crucial role in regulating the development of plant pollen, the growth of pollen tubes, seed formation and germination. For example, the loss of function of *NTT* (*AT3G57670.1*) and *ZAT4* (*AT2G45120.1*) leads to abnormal termination of pollen tube growth and affects fertilization efficiency [67]. And in *Brassica rapa* (*B. rapa*), the inhibition of *BcMF20* expression causes the complete collapse of pollen grains, directly resulting in male sterility [68].

In terms of floral organ morphogenesis, *VvC2H2* controls petal size and shape by regulating cell proliferation, while *RBE* and *JAG* are involved in sepal development. *SUP*, *MtSUP*, *AG*, and *KNU* participate in floral meristem determination and organ identity establishment. During stamen and pistil development, *OsSRO* regulates the proper formation and separation of stamens, *HvSL1* promotes stamen identity, *SE3.1* is involved in the shift to self-pollination, and *SUPP* directly regulates gene expression in embryo sac mother cells to mediate ovule proliferation. Pollen development and fertility maintenance are co-regulated by *BoC2H2*, *AtZAT4*, *BcMF20*, *AtZFP5*, and *AtZFP6*, while flowering time is modulated by factors such as Bud in maize and *IDD*. Additionally, regulators, including *RA1* and *LATE,* participate in specific aspects of floral organ development, collectively forming a regulatory network.

### 3.6. The Influence on the Formation of Fruits

C2H2 TFs, through their unique molecular structures (such as EAR inhibitory motifs) and extensive regulation of metabolic pathways, regulate developmental timing, metabolic flow, and cellular structure, thereby comprehensively controlling the formation mechanisms of fruit yield and quality [69,70]. Not only can it directly control the fruit ripening process, as one example, but specific members of *Musa ‘Hybrids*’, *C. melo*, and *S. lycopersicum* have also been proven to delay ripening, thereby affecting the post-harvest preservation and shelf life of the fruits [71,72,73]. It can also precisely control the shape and sensory quality of the fruits, such as directly influencing the determination of the fruit length of *C. sativus* [74]. Meanwhile, *CsSBS1* can promote the size of fruit spines in *C. sativus* fruits through ethylene biosynthesis mediated by *CsACO2* [75]. The fruit’s color and flavor are the product’s key characteristics. C2H2 TFs also play a central role. In *Citrus unshiu*, *CitZAT4* can enhance the orange color of the flesh by promoting carotenoid biosynthesis [76]. In *Diospyros kaki* (*D. kaki*), *DkZF1* and *DkZF2* regulate the tannin metabolism pathway, thereby influencing the astringency level of the fruit [77]. The local tissue (LT) of *S. lycopersicum* fruit is a unique jelly-like structure that is crucial for seed development and dissemination, preventing premature germination and initiating fruit ripening. *SlZFP2* can act as a regulatory factor for LT morphogenesis, controlling the occurrence of LT morphology [78]. Furthermore, the absence of certain functions may also lead to adverse phenotypes. For instance, in *A. thaliana*, the abnormal function of *WIP2* causes excessive lignification of the fruit cell walls and leads to cracking, which provides clues for maintaining the complete mechanism of fruit formation [79].

### 3.7. The Influence on the Seeds

C2H2 TFs play a central regulatory role in multiple key plant reproduction and development stages. By integrating environmental signals with internal developmental programs, they perform indispensable functions in seed germination, quality formation, and developing specialized organs such as fibers. C2H2 TFs exhibit significant environment-dependent bidirectional regulatory capabilities during seed development and germination [67,80]. The WIP expressed in embryos and suspensor organs can coordinate the direction of cell division in embryonic organs [36]. *MRPI-1* (*GRMZM2G139160_P01*) and *MRPI-2* (*GRMZM2G105224_P01*) can interact with specific transcription factors in the endosperm transfer layer, enhancing the activity of the specific promoter of endosperm transfer cells [81]. For instance, in *T. aestivum*, *TaZFP38* inhibits seed germination under drought and salt stress conditions. It promotes it under MeJA treatment, highlighting its biological function of responding to different environmental signals and flexibly adjusting the plant’s reproductive strategy [82]. At the same time, it also profoundly shapes the seeds’ characteristics. Members such as *WIP1*/*TT1* in *A. thaliana* and *Bra023223* in *B. rapa* directly determine the color of the seed coat by regulating the biosynthesis pathways of anthocyanins and flavonoids, which is an important seed appearance and quality trait [83,84,85].

Furthermore, the functions of C2H2 TFs extend to regulating various key economic traits. In *G. hirsutum*, *GhZFP8*, by coordinating a wide range of physiological processes such as photosynthesis and biomass synthesis, governs the initiation and elongation of cotton fiber cells [86,87,88]. In *T. aestivum*, the EAR motif-containing *Tipped1* (*B1*), acting as a dominant awn repressor, directly regulates the development of the awn, a spiny structure beneficial for photosynthesis and seed dispersal [89].

### 3.8. The Influence on Plant Cell Walls

C2H2 TFs directly affect the cell wall’s structural strength and chemical properties by coordinating the biosynthesis of components such as lignin and cellulose [90]. Studies have shown that C2H2 TFs play a negative regulatory role in multiple species. For example, *OsIDD2* in *O. sativa* and *PagIDD15A* in *Populus przewalskii* (*P. przewalskii*) have been confirmed to be able to inhibit the deposition of secondary cell wall or the synthesis of lignin, thereby affecting the thickening process of the cell wall [7,91]. Moreover, during the domestication of *G. max*, the *SH1* gene effectively reduces the fragmentation rate of pods by inhibiting the thickening of the secondary cell wall of the fiber cap. This mechanism provides a typical example for understanding the genetic basis of domestication traits such as seed shattering in crops [92].

### 3.9. The Influence on Plant Hormones

C2H2 TFs finely regulate the synthesis and signal transduction of various plant hormones through a bidirectional regulatory mechanism, coordinating the molecular mechanisms of plant growth, development, and environmental adaptation. In the ABA pathway, multiple C2H2 TFs, such as a potential target of casein kinase II like *AtYY1*, can bind to the promoter of *ABA REPRESSOR1* (*ABR1*), thereby exerting a negative regulatory role in ABA signaling. *G. max* plays a dual role as a negative feedback regulator in the ABA signaling pathway: its expression is induced by ABA, while it fine-tunes ABA signaling intensity by suppressing positive regulators (*ABF4*, *ABI5*, *SnRK2s*) and activating negative regulators (*ABI1*, *ABI2*), thereby maintaining a balance between stress response and growth and development [93,94]. At the same time, C2H2 TFs also deeply participate in the metabolic balance of IAA and GA. In *A. thaliana*, some members, such as *ZF2* (*AT3G19580.1*), *ZF3* (*AT5G43170.1*), and *ZAT6* (*AT5G04340.1*), located in the downstream effector layer of the JA signaling pathway, are rapidly induced by JA, and they execute JA-mediated growth inhibition by suppressing multiple aspects of the IAA signaling pathway [95]. The members of the IDD subfamily promote the synthesis and transport of IAA [96,97]. In the GA pathway, factors such as *OsZFP207* in *O. sativa* negatively regulate GA biosynthesis by directly binding to the promoter of the key GA biosynthesis gene *OsGA20ox2* (*SD1*) and repressing its transcription, thereby controlling cell elongation, plant height, and grain size. This provides a new molecular mechanism for the expression regulation of the Green Revolution gene [98].

It is worth noting that C2H2 TFs also play a significant regulatory role in the accumulation of secondary metabolites such as anthocyanins [99]. For instance, in *M. pumila*, *MdZAT5* and *MdZAT17* serve as an upstream positive regulator of the anthocyanin signaling pathway, which activates the pigment biosynthesis pathway and positively regulates pigment accumulation [100,101], while in *D. kaki*, *DkZF6* inhibits the accumulation of tannin substances [102]. What is more remarkable is that some members, such as *V. vinifera*’s *VvZFP10*, can respond to multiple hormone signals, positioned downstream of MeJA, ABA, SA, and low-temperature signaling pathways; its expression is induced by multiple hormones and stresses, and its promoter region is rich in corresponding cis-elements, thereby constituting a signaling integration node [103].

## 4. The Role of Plants in Resisting Biotic Stress

C2H2 TFs play a central regulatory role in plants’ resistance to biological invasions. They construct a synergistic defense network by activating the plant immune system and directly targeting pathogens. At the plant immune level, C2H2 TFs can rapidly respond to infection and regulate early defense signals such as reactive oxygen species (ROS) bursts and hormone networks, thereby initiating systemic resistance. For example, specific C2H2 TF alleles in *O. sativa* can confer broad-spectrum resistance to rice blast by inhibiting H_2_O_2_ degradation. While *TaZFP8-5B* in *T. aestivum* positively regulates innate immunity against Fusarium by interacting with calmodulin proteins [104,105]. In *Prunus persica*, *PpC2H2-3* promotes glutamate accumulation by transcriptionally repressing the expression of *PpGGAT1*, a key enzyme involved in glutamate metabolism, thereby not only enhancing umami taste but also improving resistance to brown rot and Drosophila infection [106]. Notably, some members, such as *MoSDT1*, exhibit complex bidirectional regulation, enhancing pathogen toxicity while strengthening plant defense, revealing their special position in the co-evolution of pathogens and hosts [107].

What is even more unique is that C2H2 TFs can directly intervene in the development of pathogens, effectively reducing their infectivity by destroying their cell wall structure [108,109]. At the same time, they serve as key hubs in the defense response of various crops such as *Solanum tuberosum* (*S. tuberosum*), *Prunus pseudocerasus*, and *Arachis hypogaea*. They can rapidly upregulate after pathogen infection and directly activate the expression of downstream disease-resistant genes [110]. These mechanisms collectively demonstrate the multi-level regulatory functions of C2H2 TFs in the plant immune system, coordinating the plant’s defense response while directly targeting pathogens, forming a highly efficient and coordinated disease-resistant mechanism.

## 5. The Role in Plant Resistance to Abiotic Stress

C2H2 TFs are a key component for enhancing the environmental adaptability of plants. They enable plants to adjust their growth and development strategies, maintain basic life activities, and minimize stress damage under non-biological stress conditions. For example, approximately 65.4% of PyC2H2 members in pear contain few introns (0–1), and this structural simplicity may facilitate rapid transcriptional responses under stress conditions [108]. By coordinating various physiological processes such as stomatal movement, reactive oxygen metabolism, and stress-related gene expression, they help plants resist multiple environmental pressures, including drought, salinity, extreme temperatures, nutrient deficiency, and heavy metal toxicity. For instance, in *P. przewalskii*, *OSIC1* mediates the rapid closure of stomata by regulating the accumulation of H_2_O_2_ in guard cells, to activate the stress resistance mechanism when exposed to abiotic stress, thereby reducing water loss [111]. Meanwhile, through our analysis, we found that most of the genes related to stress are regulated by C2H2 TFs with transcriptional inhibitory activity that contain EAR motifs. For instance, in *T. aestivum*, members such as *TaZFP23* regulate growth negatively under salt and drought stress to preserve survival resources [112]. And it has been proven to significantly enhance stress resistance in plants such as *B. napus*, *C. sativus*, *Z. mays*, and *Nelumbo nucifera*, highlighting their conservation and importance in the plant kingdom [113,114,115,116].

In this section, we emphasize that C2H2 TFs enhance the tolerance to abiotic stresses, particularly heavy metal stress, drought, flooding, salt, cold, heat stress, and mechanical stress (Table 1).

### 5.1. Reduce the Toxicity of Heavy Metals

**Figure 4:**
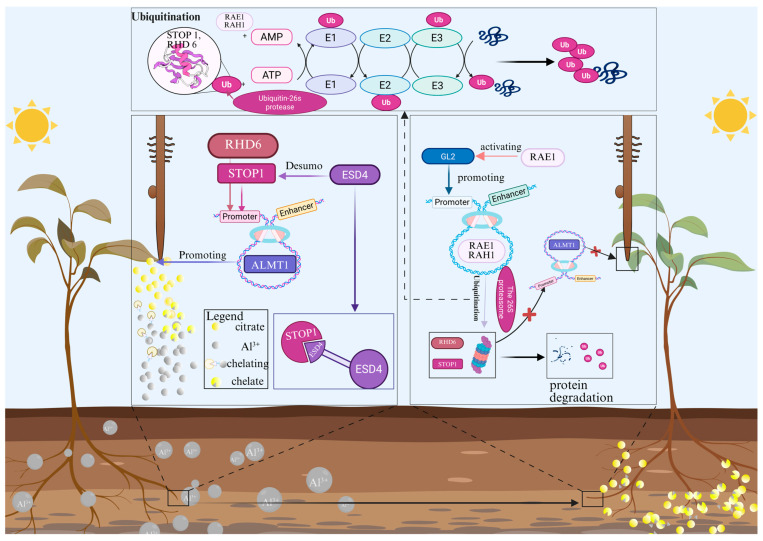
Molecular mechanism of C2H2 transcription factors in alleviating aluminum toxicity.

C2H2 TFs play a crucial role in plants’ response to maintaining the homeostasis of metal elements, and their regulatory network covers multiple physiological processes ranging from secondary metabolism to ion homeostasis. Particularly, they alleviate the toxicity of Al^3+^ through multiple mechanisms such as regulating organic acid secretion, reactive oxygen species scavenging, and secondary metabolism, enhancing the Al^3+^ resistance of plants. When there is a large number of Al^3+^ distributed in the soil, the key transcription factor represented by *STOP1* (*AT1G34370.1*) directly activates the expression of downstream aluminum tolerance genes (such as *MATE* and *ALMT1*), promoting the secretion of organic acids (such as citric acid), which then chelate with toxic Al^3+^ in the rhizosphere for external detoxification. This pathway is highly conserved in various species, including *A. thaliana*, *G. max*, *N. tabacum*, *Cajanus cajan*, and *G. hirsutum* [140,141,142]. Figure 4 presents all the pathways by which plants alleviate Al^3+^. Firstly, the activity of *STOP1* is strictly regulated by post-translational modifications. The small ubiquitin-like modifier (SUMO) protease ESD4 can enhance the binding ability of *STOP1* to the *ALMT1* promoter through SUMO removal, thereby positively regulating the aluminum resistance of plants [143,144]. Secondly, the function of *STOP1* is integrated into a broader protein interaction module (such as RAE1-GL2-STOP1-RHD6), which jointly regulates the secretion of citrate [145]. Furthermore, in *O. sativa*, the *ART1* gene performs multi-level aluminum detoxification from the inside of the cell to the outside by interacting with the *STAR1* [146]. In addition to the above-mentioned core pathways, C2H2 TFs also enhance aluminum tolerance through other mechanisms. For instance, *GsGIS3* can reduce aluminum accumulation in the root system [145]. Members of *Abies fabri* alleviate aluminum toxicity by coordinately regulating secondary metabolic pathways, such as flavonoids [147].

Apart from aluminum toxicity, C2H2 TFs can also mediate the tolerance of plants to different heavy metals through various molecular mechanisms. Under Mn^2+^ stress, C2H2 TFs in *Stylosanthes hamata* (*S. hamata*) regulate secondary metabolic pathways through transcription, promoting the synthesis of compounds such as phenols and tannins to enhance tolerance [148]. For Ni stress, *ZAT11* (*AT2G37430.1*) in *A. thaliana* exhibits a unique negative regulatory function, inversely regulating the tolerance level of the plant [149]. In Cd stress, the C2H2 TFs in *Koelreuteria paniculata* can rapidly respond to stress signals and initiate expression. These findings collectively reveal that C2H2 TFs have extensive functions in the response to heavy metal stress, and their regulatory network covers multiple physiological processes ranging from secondary metabolism to ion homeostasis [150].

Under Al^3+^ stress, the *STOP1* is stabilized and activates downstream gene expression. *RAE1* mediates the degradation of *STOP1* through the ubiquitination pathway, whereas *ESD4* promotes *STOP1* stability via deSUMOylation modification. Stabilized *STOP1* directly activates the expression of the malate transporter gene *ALMT1*, promoting malate secretion from the root tip. Malate chelates Al^3+^ to form non-toxic complexes, thereby alleviating Al^3+^ toxicity. Meanwhile, *RHD6* is involved in root hair development, and *GL2* may participate in epidermal cell differentiation.

### 5.2. Adapt to Drought Stress

**Figure 5:**
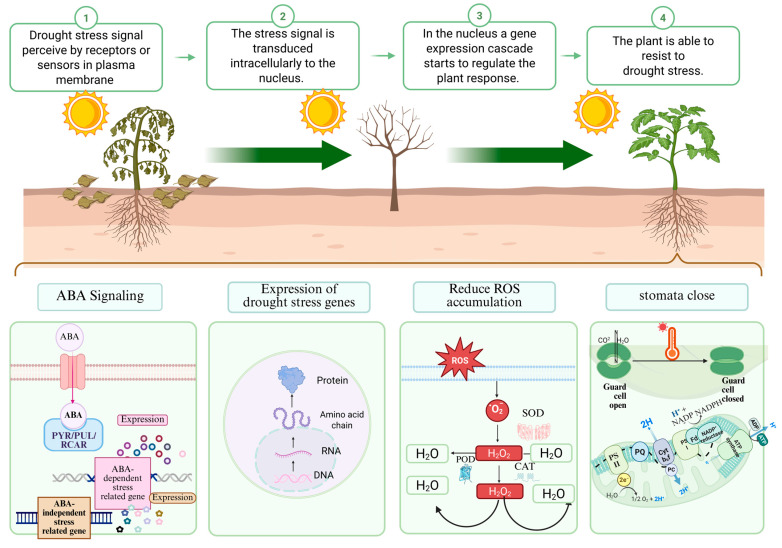
Schematic model of the regulatory mechanism of C2H2 TFs in response to drought stress.

C2H2 TFs play a central role in plant drought resistance, and their mechanism of action encompasses various physiological levels ranging from stomatal movement to metabolic reprogramming. As shown in Figure 5, these regulatory mechanisms can be systematically summarized into four core aspects: precise regulation of stomatal movement, maintenance of ROS homeostasis, regulation of the expression of drought-related genes, and integration of plant hormone signals. It can not only enhance the drought resistance of plants, but also increase their tolerance to drought. When plants respond to drought signals, factors such as *TaZAT8-5B* in *T. aestivum* can be able to quickly close the pores to reduce water loss [34,151]. *BcZAT12* in *S. lycopersicum*, *MdZAT10* in *M. pumila*, *OsDRZ1* in *O. sativa*, *Styphnolobium japonicum*, and *AtSTZ1* and *AtZAT18* in *A. thaliana* enhance the antioxidant defense system to alleviate the accumulation of ROS, which enhances the drought resistance of plants [123,125,139,152]. Meanwhile, C2H2 TFs can regulate the secondary metabolites of plants to enhance their drought resistance. For example, *Ophiopogon japonicus* (*O. japonicus*) enhances drought tolerance by promoting flavonoid synthesis, while in the aspect of signal integration, C2H2 TFs can transmit stress signals through both ABA-dependent pathways and ABA-independent pathways (such as *PuZFP103*) [21,153]. For example, *ZFP151* in *O. sativa* enhances drought tolerance by directly activating the expression of *NCED4*, a rate-limiting gene in the ABA biosynthesis pathway [121].

From *AtSTZ1* in *A. thaliana* to *OsDRZ1* in *O. sativa*, from *TaZFP1B* in *T. aestivum* to *PtrC2H2.2-6* in *Populus trichocarpa*, these factors exert their functions through unique molecular pathways in their respective species [120,127,130,137,154]. It is particularly noteworthy that the bidirectional regulatory characteristic of this family—within the same family, there are positive regulatory factors, such as *TaZFP1B*, and negative regulatory factors, such as *TaZFP21* and *PtrC2H2.2-6*. This precise balance mechanism ensures that plants can respond appropriately according to the intensity of stress and environmental conditions [128].

C2H2 TFs regulate drought adaptation primarily through the following four pathways: ① activating the ABA signaling pathway (mediated by the PYR/PYL/RCAR receptor complex); ② inducing the expression of drought stress-related genes; ③ reducing reactive oxygen species (ROS) accumulation; ④ regulating stomatal closure (promoting the transition of guard cells from an open to a closed state to reduce water loss).

### 5.3. Deal with Salt Stress

C2H2 TFs’ salt stress responses vary among species, showing specific conservation and significant functional specificity. Studies have shown that from ancient *Rhodophyta* to advanced terrestrial plants, C2H2 TFs are all involved in the adaptation process to salt stress, and the mechanisms of their actions exhibit rich diversity [155].

#### 5.3.1. Enhance Stress Resistance Capability

At the molecular mechanism level, C2H2 TFs enhance plants’ abilities to resist salt stress through multiple pathways. For instance, under salt stress, C2H2 TFs rapidly regulate ion homeostasis. In *Medicago truncatula*, *MtZPT2-2* reduces Na^+^ unloading in the xylem to limit the transport of sodium ions to the aboveground parts [100,156,157,158]. It can also activate the synthesis pathway of osmotic adjustment substances to maintain cellular osmotic balance, and at the same time, regulate the reactive oxygen species metabolism system to maintain redox homeostasis [48,130,159,160,161,162,163,164]. It is worth noting that some members, such as *GhDi19-3* and *GhDi19-4* in *G. hirsutum*, enhance the negative regulation of oxidative damage to enhance salt tolerance, demonstrating the complexity of the functions of C2H2 TFs [165].

#### 5.3.2. Increase in Tolerance Level

It is worth noting that C2H2 TFs have a dual function in regulating plant hormones and enhancing plant salt tolerance [100,166,167]. For example, under salt stress conditions, in *Malus pumila*, *MdZAT5* and *MdZAT17*, although both belong to C2H2 TFs, exert negative and positive regulatory effects, respectively. This fine division of labor ensures that plants can respond appropriately according to the intensity of stress [100]. Recent studies have also found that in the process of using computer simulation to assist in screening superior alleles, *CqZAT4* and *CqZAT6* in *Chenopodium quinoa* are closely related to improving root structure and water retention under salt stress. This provides new ideas and targets for cultivating salt-tolerant crop varieties using C2H2 TFs [168].

### 5.4. Deal with Temperature Stress

When C2H2 TFs are subjected to temperature stress, they can regulate cold tolerance through the conserved ICE-CBF-COR pathway, and also cope with high-temperature stress through mechanisms such as heat shock protein regulation and redox homeostasis maintenance, providing important theoretical basis and genetic resources for crop stress resistance breeding [103,169,170,171].

Under low-temperature stress, the C2H2 TFs mainly regulate cold tolerance through the ICE-CBF-COR core signaling pathway [172]. In *Musa* ‘*Hybrids*’, *MaC2H2-2* and *MaC2H2-3* participate in the cold stress response by inhibiting *MaICE1* transcription. By suppressing excessive responses to manage the stress response, this belongs to the early adaptation strategy [173]. Meanwhile, in *O. sativa*, *OsIDD3/ROC1* directly targets the DREB/CBF1 cis-element to activate downstream cold-resistant genes, directly activating the defense program to enhance survival capabilities in low temperatures falls [174]. Besides this main pathway, some members, such as *ZFP245* and *SCOF-1* in *G. max*, function through an ABA-independent pathway [175]. Other members, such as *PeSTZ1* in *Populus euphratica* and *NtZAT12* in *Nymphaea tetragona*, enhance cold resistance by increasing the activity of antioxidant enzymes and accumulating osmotic regulatory substances like soluble sugars [176,177]. The latest research has found that vitamin E is expressed more strongly when plants are under extreme cold stress. At this time, C2H2 TFs are highly enriched in this pathway to enhance the cold resistance of plants [178].

During the response to high-temperature stress, C2H2 TFs enhance heat tolerance through various molecular strategies [179]. In *S. lycopersicum*, *ZAT12* upregulates the expression of heat shock proteins Hsp17.4 and Hsp21, while also enhancing the ability to remove reactive oxygen species to alleviate oxidative damage induced by heat shock [180]. In *C. annuum*, *CaZFN830* can positively induce the expression of heat tolerance-related genes [181]. Meanwhile, in *O. sativa*, *NAT1* enhances heat tolerance by increasing wax deposition. [182].

### 5.5. Others

C2H2 TFs exhibit unique regulatory functions in plants during responses to environmental stresses such as mechanical stress and atmospheric particulate matter (PM) pollution [183]. Studies have shown that C2H2 TFs can sense mechanical signals and participate in corresponding adaptive regulation. For instance, *PtaZFP2* in *P. przewalskii* rapidly upregulates its expression after the stem is subjected to bending and other mechanical stimuli. As a negative regulatory factor, it inhibits the excessive response of the plant to mechanical signals, thereby maintaining normal growth and development [183,184]. Moreover, C2H2 TFs also adapt plants to atmospheric pollution by promoting the accumulation of osmotic regulatory substances such as proline and soluble sugars, enhancing the cell’s resistance to pollution stress [185]. Under phosphate-deficient conditions, *SlSTOP1* in tomato promotes primary root elongation by activating the citrate transporter *SlFRDL1*, which drives citrate exudation from roots, chelates Fe^2+^ in the root tip to form Fe-citrate complexes, thereby reducing ROS accumulation [186].

## 6. The Biosynthesis of Secondary Metabolites in Plants

C2H2 TFs can influence the accumulation of metabolites with important biological functions and economic value by precisely regulating multiple biosynthetic pathways.

**Figure 6:**
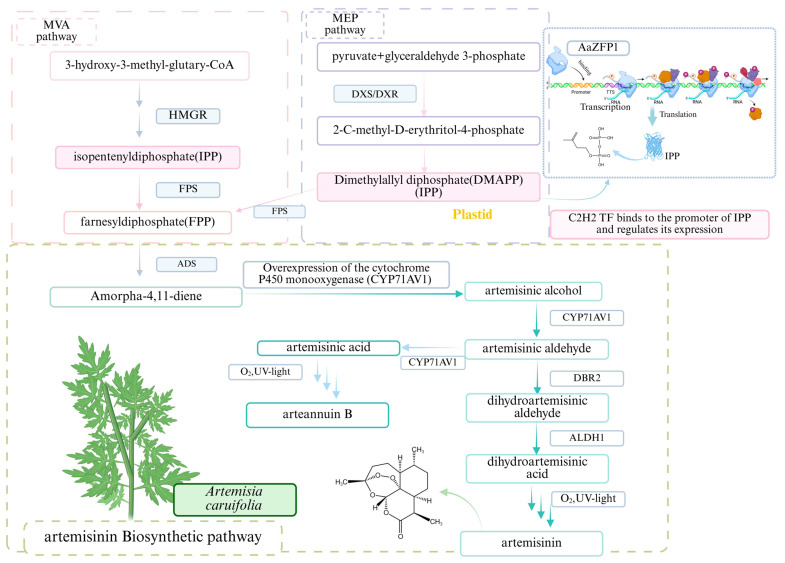
Involvement of C2H2 TFs in regulating artemisinin biosynthesis. In the MVA pathway, key enzymes including HMGR, FPS, ADS, and CYP71AV1 catalyze sequential reactions converting HMG-CoA into artemisinin. C2H2 TFs may participate in the transcriptional regulation of artemisinin biosynthesis by modulating the expression of these key enzyme genes. Oxygen and UV light are also involved in the later stages of the biosynthetic process.

As shown in Figure 6, in the biosynthesis pathway of artemisinin, *AaZFP1* positively regulates the synthesis of this rare terpenoid compound by directly binding to the promoter of the key enzyme gene *AaIPPI1*, demonstrating the direct regulatory role of C2H2 TFs in the biosynthesis of medicinal plants [187].

In addition to terpene metabolism, C2H2 TFs are also widely involved in regulating phenylpropanoid and flavonoid compound synthesis [131,188,189,190,191,192]. C2H2 TFs can orchestrate the interplay between hormone and stress signaling to shape the expression bias of the flavonoid pathway in *Safflower* [193]. In *Capparaceae*, *C2H2-17* has been identified as the central regulatory factor of the phenylpropanoid metabolic network, showing a strong co-expression relationship with multiple structural genes [194]. In *G. max*, *GmZFP7* regulates the expression of isoflavone synthase and flavanone hydroxylase coordinately, simultaneously increasing the metabolic flux of the phenylpropanoid pathway and promoting the accumulation of isoflavones [195]. Moreover, in the aspect of polysaccharide synthesis, C2H2 TFs regulate the expression of sucrose synthase and sugar transporters and other related genes, affecting the content and composition of polysaccharides and sugar components in various plants [196,197,198]. This provides key clues for deciphering the molecular mechanism of synthesizing special metabolites in plants and lays a theoretical foundation for improving the yield of target active components through synthetic biology strategies.

## 7. Discussion

TFs are key factors that regulate gene expression. By interacting with DNA, RNA, or other proteins, they control the initiation, termination, and entire process of gene transcription. Therefore, studying the functions and mechanisms of TFs in plants holds significant theoretical and practical importance [199]. C2H2 TFs have been proven to play an indispensable core role in the plant life cycle. They form a complex and precise regulatory network that links growth and development, environmental responses, and metabolic regulation. C2H2 TFs play a crucial role in growth and development at various stages, such as seed germination, root architecture, leaf morphology, and flower organ development. Their functions are often achieved by integrating hormone signaling pathways (such as IAA, GA, ET, and ABA) to control cell division, differentiation, and organ formation precisely. In response to stress conditions, whether it is non-biological stress such as drought, salinity, extreme temperatures, or biological stress such as pathogen invasion, C2H2 TFs can act as critical regulators, rapidly initiating defense gene expression, maintaining ROS homeostasis, and regulating the opening degree of stomata, thereby endowing plants with strong environmental adaptability. Moreover, C2H2 TFs are also the “key integrator” of plant secondary metabolites, directly regulating the biosynthesis of important active components such as terpenoids (e.g., artemisinin), phenylpropanoids (e.g., flavonoids), and alkaloids. This profoundly reveals their core position in plant chemical defense and quality formation.

## 8. Conclusions

Although significant progress has been made in understanding the functions of plant C2H2 TFs, this field still faces numerous challenges and opportunities. Current research is largely based on transcriptomic analyses of mixed tissues or specific developmental stages, which limits the precise dissection of C2H2 member functions in specific cell types and in response to stresses. Furthermore, functional characterization remains primarily concentrated in a few model species, leaving the functions of the vast majority of family members unknown. Whole-genome duplication events have led to the expansion of C2H2 TFs, potentially resulting in functional redundancy, which makes it difficult to effectively uncover their true functions using traditional genetic approaches. Research on pseudogenization within this family is almost nonexistent. Although evolutionary analyses (e.g., low selection pressure in Chinese cabbage [6]) suggest the possibility of extensive pseudogenization among C2H2 TFs, there is a lack of systematic attention to the types, mechanisms, and the question of whether certain “pseudogenized” forms still possess latent regulatory functions (e.g., as regulatory pseudogenes).

Therefore, future research should focus on breakthroughs in the following areas: integrating single-cell sequencing technologies to systematically map the spatiotemporal expression atlas of C2H2 TFs during specific developmental phases and in response to complex stress conditions; combining proteomics and multi-omics analyses to reveal how C2H2 TFs synergize with other transcription factors and epigenetic modifiers in specific tissues or cell types to form complete regulatory modules; deeply investigating the bidirectional regulatory mechanisms of the same member under different scenarios, elucidating how interacting proteins and post-translational modifications mediate their functional transitions; systematically identifying pseudogenization events and their biological significance, exploring whether “pseudogenized” forms still possess latent regulatory functions; and enhancing the exploitation of élite allelic variation in crops to synergistically optimize the “stress tolerance-yield-quality” trilemma through gene editing or marker-assisted breeding strategies.

## Figures and Tables

**Table 1:** Abiotics stress search progress of C2H2 TFs in plants.

Gene	Species	Type of Abiotic Stress	Reaction Type	Function	Ref.
*OsZOS2-19*	*Oryza sativa*	low temperature	negative	Negatively regulates low temperature response and ABA signaling genes (*OsPGL12*, *OsWRKY71*), leading to reduced ROS scavenging capability, increased membrane damage, and decreased soluble sugar content under low temperature conditions.	[117]
*OsZFP252*		salt, drought	positive	Enhances the plant’s osmotic adjustment capacity and antioxidant enzyme activities by increasing the accumulation of free proline and soluble sugars.	[118]
*OsZFP245*		drought	positive	Enhances the activities of ROS-scavenging enzymes such as SOD and POD, thereby improving tolerance to H_2_O_2_.	
*OsDRZ1*		drought	positive	The accumulation of more free proline and less ROS enhanced the activity of antioxidant enzymes and upregulated drought-related genes.	[119]
*OsDi19*		drought	positive	Interacts with drought response proteins in vitro and in vivo.	[120]
*OsZFP151*		drought	positive	*ZFP151* directly binds to the promoter of *NCED4*, a rate-limiting gene in the abscisic acid (ABA) biosynthetic pathway and transcriptionally activates its expression.	[121]
*AtZAT6*	*Arabidopsis thaliana*	Salt, drought, low temperature	positive	Directly binds to the TACAAT motif in the promoters of target genes, activating salicylic acid (SA)-related genes (*EDS1*, *PAD4*, *PRs*) and CBF cold-responsive genes, thereby promoting the accumulation of SA and reactive oxygen species (ROS).	[122]
*AtSTZ1*		drought	positive	Higher proline, chlorophyll, soluble sugars and enhanced ROS scavenging enzyme activity.	[123]
*AT2G47270*		drought	positive	The transpiration of soil water FTSWc was decreased and the transpiration rate after FTSWc was increased. This suggests that this gene is involved in the regulation of stomatal closure, delaying the moment of stomatal closure and FTSW in plants	[124]
*AtZAT18*		drought	positive	Less leaf water loss, lower reactive oxygen species (ROS) content, higher leaf water content and higher antioxidant enzyme activity positively regulated stress response genes and hormone signaling related genes, interacting with drought response proteins	[125]
*TaZFS 21*	*Triticum aestivum*	drought	positive	Positively involved in ABA-dependent gene regulatory pathways.	[126]
*TaZFS 22*			positive		[126]
*TaZFS 23*			positive		[126]
*TaZFS 33*			positive	Positively involved in ABA-dependent gene regulatory pathways, and positively regulated corresponding drought genes.	[126]
*TSB 34*			positive		[126]
*TaZFS 37*			positive		[126]
*TaZFS1B*			positive	Upregulates different oxidative stress response genes, making plants more drought tolerant.	[127]
*TaZFS21*			negative	Overexpressed plants showed low activity of ROS-related scavenging enzymes and increased sensitivity to drought and ABA.	[128]
*TaZAT8-5B*			positive	Increased Pro content and SOD activity, decreased stomatal pore size to reduce water loss, and induced expression of related genes, alleviated MDA accumulation induced by drought stress and improved drought tolerance.	[34]
*ZxZF*	*Zygophyllum xanthoxylum*		positive	Regulate stomatal opening to increase intercellular CO_2_ concentration, and enhance photosynthesis in response to drought stress by increasing chlorophyll content, photosynthetic performance index and photochemical efficiency.	[129]
*MsZFS1*	*Medicago sativa*		positive	The expression of drought-induced response marker genes (MtCOR47, MtRAB18, MtP5CS and MtRD2) was enhanced.	[130]
*BcZAT12*	*Solanum lycopersicum*	drought	positive	Significantly reduce the amount of electrolyte leakage, increase the relative water content, increase the level of proline, reduce ROS accumulation and inhibit oxidative stress by increasing the activity of deoxygenase (CAT, SOD, APX, ADDIN EN.CITE and other enzymes).	[128,129]
*PuZFS103*	*Populus ussuriensis*		positive	Responds positively to ABA signaling pathways ADDIN EN.CITE.	[130]
*SrC2H2.2i-Q.19*	*Stevia rebaudiana Bertoni*		positive	Significantly expressed under drought stress. It was significantly expressed under drought stress.	[21]
*SrC2H2.2i-Q.23* (*SrZAT18*, *AT3G53600*)			Double regulation	Higher leaf water content and antioxidant enzyme activity, increased drought tolerance, sensitivity to ABA, and decreased salt tolerance.	
*OjC2H2 TFs*	*Ophiopogon japonicus*		positive	Regulates the biosynthesis of flavonoid compounds.	[131]
*SCOF1*	*Glycine max*	low temperature	positive	Enhances the expression of downstream cold-responsive genes through an ABA-dependent signaling pathway, thereby improving plant cold tolerance.	[118]
*OSIC1*	*Populus alba var. pyramidalis*	salt, drought	positive	Induced by salt, drought, and ABA, it directly targets and activates the PalCuAOζ gene, promoting H_2_O_2_ accumulation in guard cells, thereby inducing stomatal closure to reduce water loss. Its transcriptional activity can be enhanced by phosphorylation by PalMPK3.	[9]
*PeZFP38*	*Populus euphratica*	salt	positive	Is strongly induced by salt stress, and enhances antioxidant capacity by integrating ABA signaling and the ROS scavenging system (increasing SOD activity), thereby alleviating oxidative damage and improving salt tolerance.	[8,132]
*PpZAT10*	*Prunus persica*	low temperature	negative	Enhances vacuolar invertase (VIN) activity, leading to increased sucrose hydrolysis, thereby exacerbating chilling injury and negatively regulating cold tolerance in peach.	[133]
*PtrZAT12*	*Poncirus trifoliata*	low temperature	positive	As a direct target of CBF1, its overexpression enhances cold tolerance in transgenic tobacco by promoting ROS scavenging.	[134]
*MhZAT10*	*Malus honanensis*	drought, low temperature, infiltration	positive	Direct target of DREB2A, Positively regulate tolerance to both drought and cold stresses via activating downstream target genes MhWRKY31, MhMYB88, and MhMYB124. Directly binds to the promoters of antioxidant enzyme genes (MhMSD1, MhAPX3a, MhCAT1) and activates their transcription, thereby enhancing ROS scavenging capacity in response to osmotic stress.	[135,136]
*PeSTZ1*	*Populus euphratica*	salt	positive	By directly regulating the expression of APX2, it enhances ROS scavenging capability, improving salt tolerance.	[45]
*PtrC2H2.2-6*	*Populus trichocarpa*	drought	negative	Directly binds to the CACT motif in the promoters of PtrCYP86A7 and PtrCYP86A8, repressing their expression, thereby negatively regulating cutin and wax biosynthesis and reducing leaf water retention. The PtrPPK1 kinase can interact with it and promote its degradation, relieving this inhibition.	[137]
*MdZAT5*	*Malus domestica*	drought	positive	Directly binds to the promoters of drought-responsive genes (such as MdRHA2a, MdLEA14) to activate their expression, and interacts with MdHYL1 to regulate the biogenesis of drought-responsive microRNAs, thereby positively modulating root development and hydraulic conductivity.	[138]
*MdZAT10*		drought	negative	The increased water loss reduced the expression level of APX2, and the excessive accumulation of MDA and ROS in apples enhanced the sensitivity to drought stress.	[139]

## Data Availability

No new data were created or analyzed in this study. Data sharing is not applicable to this article.

## Abbreviations

The following abbreviations are used in this manuscript:

ZFS	Zinc finger structures
C2H2 TFs	Cys2-His2-type zinc finger transcription factors
ROS	Reactive Oxygen Species
EPSPS	5-enolpyruvylshikimate-3-phosphate synthase
SUP	SUPERMAN
WIP	Wound-induced protein
JAG	JAGGED
EAR	Ethylene response element-binding factor-related
GA	Gibberellin
CTK	cytokinin
SIM	SIAMESE
H	HAIR
SH	SPARSE HAIR
CPC	CAPRICE
PCD	programmed cell death
FIS	Fertilization-independent seed gene
SCW	secondary cell wall
N	Nitrogen
P	Phosphorus
PT	phosphate transporter
AE	antioxidant enzymes
NUE	nitrogen use efficiency
Mg	magnesium
Ca	calcium
Zn	zinc
Fe	iron
Cu	copper
Al	aluminum
Mn	manganese
Ge	chromium
SUMO	small ubiquitin-like modifier
Pro	proline
FTSWc	fraction of soil water transpiration
INS	The inositol and its derivatives
RFO	raffinose oligosaccharide family
PAN	phosphatase
CAN	calcineurin
DREB	dehydration reaction element-to-binding protein
NR	no rhizomes at the seedling stage
